# Quantitative Assessment of Lung Volumes and Enhancement in Patients with COVID-19: Role of Dual-Energy CT

**DOI:** 10.3390/diagnostics13061201

**Published:** 2023-03-22

**Authors:** Giovanni Foti, Chiara Longo, Niccolò Faccioli, Massimo Guerriero, Flavio Stefanini, Dora Buonfrate

**Affiliations:** 1 Radiology Department, IRCCS Sacro Cuore Don Calabria Hospital, 37024 Negrar di Valpolicella, Italy; chiara.longo@sacrocuore.it; 2 Radiology Department, Verona University Hospital, 37129 Verona, Italy; niccolofcc@yahoo.com; 3 Clinical Research Unit, IRCCS Sacro Cuore Don Calabria Hospital, 37024 Negrar di Valpolicella, Italy; massimo.guerriero@univr.it; 4 Department of Emergency Medicine, IRCCS Sacro Cuore Don Calabria Hospital, 37024 Negrar di Valpolicella, Italy; flavio.stefanini@sacrocuore.it; 5 Department of Infectious Tropical Diseases and Microbiology, IRCCS Sacro Cuore Don Calabria Hospital, 37024 Negrar di Valpolicella, Italy; dora.buonfrate@sacrocuore.it

**Keywords:** COVID-19, dual-energy CT, lung perfusion, lung volumes

## Abstract

Dual-energy computed tomography (DECT) has been used for detecting pulmonary embolism, but the role of lung perfusion DECT as a predictor of prognosis of coronavirus disease 2019 (COVID-19) has not been defined yet. The aim of our study was to explore whether the enhancement pattern in COVID-19+ patients relates to the disease outcome. A secondary aim was to compare the lung volumes in two subgroups of patients. In this observational study, we considered all consecutive COVID-19+ patients who presented to the emergency room between January 2021 and December 2021 with respiratory symptoms (with mild to absent lung consolidation) and were studied by chest contrast-enhanced DECT to be eligible. Two experienced radiologists post-processed the images using the “lung-analysis” software (SyngoVia). Absolute and relative enhancement lung volumes were assessed. Patients were stratified in two subgroups depending on clinical outcome at 30 days: (i) good outcome (i.e., discharge, absence of clinical or imaging signs of disease); (ii) bad outcome (i.e., hospitalization, death). Patient sub-groups were compared using chi-square test or Fisher test for qualitative parameters, chi-square test or Spearman’s Rho test for quantitative parameters, Students’ *t*-test for parametric variables and Wilcoxon test for non-parametric variables. We enrolled 78 patients (45M), of whom, 16.7% had good outcomes. We did not observe any significant differences between the two groups, both in terms of the total enhancement evaluation (p = 0.679) and of the relative enhancement (p = 0.918). In contrast, the average lung volume of good outcome patients (mean value of 4262 mL) was significantly larger than that of bad outcome patients (mean value of 3577.8 mL), p = 0.0116. All COVID-19+ patients, with either good or bad outcomes, presented similar enhancement parameters and relative enhancements, underlining no differences in lung perfusion. Conversely, a significant drop in lung volume was identified in the bad outcome subgroup eligible compared to the good outcome subgroup.

## 1. Introduction

Lung imaging has become an important diagnostic tool for management of the coronavirus disease 2019 (COVID-19) [1,2,3,4,5,6]. Several imaging characteristics have been described at diagnosis, including focal ground-glass opacities, proximal and distal pulmonary vessel dilatation and tortuosity, mainly within, or around, the areas of lung opacities [7,8].

Many studies have addressed the pathophysiology of the thrombotic events occurring in patients with COVID-19, unveiling that a prominent role is played by the cytokine cascade [9,10,11,12]. The latter seems to trigger micro-embolic phenomena that reduce pulmonary vascularization, inducing a ventilation–perfusion mismatch and, together with the purely inflammatory and infectious alterations, aggravate the respiratory picture [13,14,15,16].

The predominant imaging pattern is a ground-glass opacification with occasional consolidation in the peripheries, without pleural effusions and lymphadenopathy. The ground-glass opacities turn into consolidation and subsequent resolution of the pneumonia. Ground-glass and consolidative opacities visible on computed tomography (CT) are sometimes undetectable on chest radiography, suggesting that CT is the most sensitive imaging modality for investigation [17]. Among CT findings observed, there was also an increased perfusion of the lungs in the proximal areas of lung opacity, decreased areas of peripheral perfusion and a dilatation of pulmonary vessels in areas of parenchymal abnormality [8]. Poschenrieder et al. demonstrated that opacifications in patients with severe COVID-19 pneumonia can be both hypoperfused and hyperperfused [16].

Dual-energy computed tomography (DECT) has been used for the identification of macroscopic pulmonary embolism and related pulmonary perfusion defects [13,18,19,20,21,22]. The main advantages of DECT are the possibility to obtain virtual monoenergetic images to improve vascular contrast, thus allowing the evaluation of the pulmonary arteries, and the identification of regional lung perfusion defects on dedicated maps of iodine concentration [19]. In particular, Foti et al. identified that venous-phase DECT sensitivity and specificity in diagnosing pulmonary embolism were 90% and 100%, respectively [18]. In addition, data have shown that lung perfusion imaging can be used to identify hypoperfused areas in the lungs. Both 2D and 3D perfusion maps can be assessed qualitatively or quantitatively by analyzing DECT numbers generated from the scanner.

Nevertheless, there are no papers focusing on the quantitative assessment of lung perfusion in the early stages of COVID-19 disease.

A few studies have also been conducted to explore the value of lung volume in COVID-19 patients, demonstrating that COVID-19 patients have a significantly reduced lung volume with increased density and mass [23,24]. Iwasawa et al. found that secondary lobes in the crazy-paving pattern were smaller to unaffected lungs, indicating that the lesions of COVID-19 pneumonia are accompanied with a local volume loss. the authors hypothesized that the volume loss might be due to an alveolar collapse [23]. This study included six patients only, and evaluated the CT lung volume and the predicted total lung capacity [23]. In another study, Shi et al. compared the lung volumes of 3389 COVID-19+ versus 3300 non-COVID-19 patients. The authors found that COVID-19+ patients had a significantly reduced lung volume, with an increased density and mass; moreover, the infections tended to be present bilaterally at lower lobes [22].

In these settings, perfusion or volume abnormalities identified in the early stage of lung involvement, in the absence of significant alveolar or interstitial lung involvement, could represent an imaging prognostic indicator to be used for the stratification of high-risk versus low-risk patients.

The main purpose of this study was to investigate whether contrast-enhanced DECT used in the assessment of lung perfusion in COVID-19+ patients can have a role to predict patient prognosis. A secondary aim was the evaluation of the lung volumes in two subgroups of patients (as defined in the following paragraph).

## 2. Materials and Methods

### 2.1. Setting and Participants

In this observational case-control study, we evaluated all consecutive COVID-19+ patients presenting to the emergency room between January and December 2021, and needing CT examination due to respiratory distress symptoms or elevated D-dimer. This single-center study was approved by the institutional review board and all patients provided informed consent (Clinical Trial Registration No. 6176; Clinical Research Ethic Committee no. 3106; Ethics Committee of the Provinces of Verona and Rovigo).

Inclusion criteria were: mild respiratory symptoms (including cough, sneezing, sore throat, breathlessness, tight chest or wheezing, high temperature), availability of contrast-enhanced DECT at their admittance, eGFR > 25 mL/min/m^2^, age over 18 years. All patients were tested for COVID-19 with polymerase chain reaction (PCR) analysis of oropharyngeal swab upon presentation to the emergency room.

Exclusion criteria were: macroscopic pulmonary embolism (considered as thrombi in the first and second grade bifurcations of pulmonary artery vessels), severe pneumonia (alveolar consolidation found at physical examination or more than 20% of involvement found at X-rays performed at the emergency room, according to chest X-ray scoring system [25]) or severe comorbidities (including patients with unfavorable outcomes caused by conditions other than COVID-19).

Patients were followed up at 1 month after the first evaluation, through clinical and physical evaluation. In most cases, chest X-rays were also performed at follow up, and in some cases, a CT was done (if there were any suspected findings at the physical examination). For study purposes, we stratified patients into two subgroups in relation to the clinical/radiological outcome: (i) good outcome (i.e., no need for hospitalization, resolution of symptoms or discharged with mild symptoms); (ii) bad outcome (i.e., worsening or persistence of severe symptoms, need for hospitalization in general care unit or in intensive care, decease).

### 2.2. Imaging Acquisition

CT examinations were performed with a third-generation 384-slice dual-source CT scanner (Somatom^®^ Definition Force, Siemens Healthineers, Enlargen, Germany). For all patients, images were acquired in full inspiration and a single body-weight-adapted administration of iodine contrast agent (1 mL/kg, Iomeron 350, Bracco, Milan, Italy) was performed using a dual-syringe power injector (Medrad Stellant, Bayer Healthcare, Indianola, PA, USA) at a flow rate of 2.5 ± 0.5 mL/s, followed by a 40 mL saline flush injection. DECT scans were acquired using a bolus tracking technique with ROI in the right ventricle. The monitoring time during the injection was 10 s, DECT was imaged with 9 s delay (after the threshold of 150 HU, with an average delay of 20 s from the injection of contrast). The scanning parameters were as follows: tube A 100 kV; tube B Sn 150 kV with a reference tube current time product of 190 mAs for tube A and 95 mAs for tube B; a ratio of 0.8 (tube A:B). Virtually blended 120 kV mixed images were obtained for clinical reading with collimation of 0.6 mm × 128, gantry rotation time of 0.5 s, pitch of 0.8 and average scanning time of 2.8 s. Automated attenuation-based tube current modulation (CARE dose 4D; Siemens Healthineers, Enlargen, Germany) was used.

### 2.3. Imaging Post-Processing

Two radiologists (with 15 and 4 years of experience), in consensus, post-processed all the imaging; any disagreement was resolved by consensus. They assessed pulmonary volumes (right versus left lung; upper, middle and lower right lobe; and upper, middle-lingular, lower left lobe). In addition, absolute and relative enhancement were calculated (adjusting the density values by positioning a ROI in descending aorta). Soft tissue kernel (Qr32; thickness 1 mm; increment 1 mm) 100 kVp and 150 kVp set images were used in an offline workstation (SyngoVia^®^ VB20; Siemens Healthineers, Enlargen, Germany). The monoenergetic (MEI+) application was used to optimize the visualization of contrast material within arterial vessels and rule out macroscopic embolism (consensus reading). The “lung analysis application” was used to generate lung perfusion images.

The lung volume was divided into right and left volume and then each lung was divided into three partitions, approximately corresponding to the anatomic lobes: partition 1 = right upper lobe, partition 2 = right middle lobe, partition 3 = right lower lobe, partition 4 = left upper lobe, partition 5 = lingula, partition 6 = left lower lobe. Therefore, a total of 566 partitions were evaluated in 78 patients enrolled in the study.

### 2.4. Statistical Analysis

For the purpose of this study, participants were divided according to their clinical outcome (good versus bad outcome). For each patient, a quantitative assessment of lung volumes and perfusion values was performed. The parameters in the good outcome patient subgroup were compared with those in the other subgroup.

Normal distribution of the data was evaluated using the Kolmogorov–Smirnov test. Estimated parameters were reported with the 95% confidence intervals using the exact method. Continuous variables were described with a mean and standard deviation (SD), while range and categorical variables were measured with a percentage distribution.

For the correlation between variables, we used chi-square test or Fisher test for qualitative parameters, chi-square test or Spearman’s Rho test for quantitative parameters, Students’ *t*-test for parametric variables and Wilcoxon test for non-parametric variables. Multivariate logistic regression models were used to investigate the connection between dichotomous dependent variables in relation to the patient’s analysis.

The statistical significance level was fixed at 5% and data analysis was performed using SAS software version 9.4.

## 3. Results

**Figure 1:**
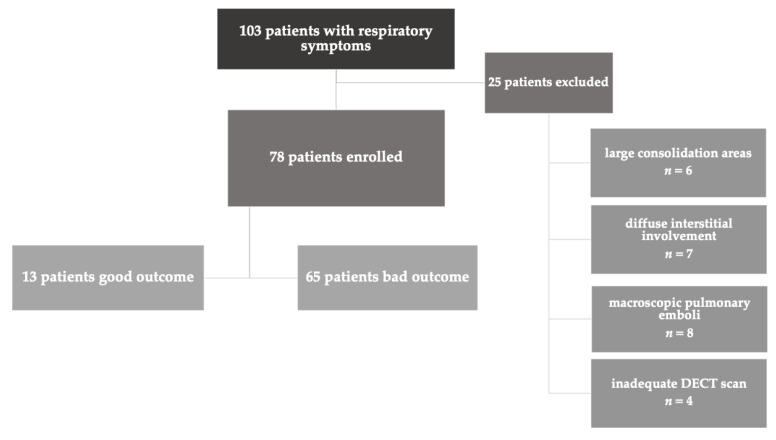
Flowchart of participants included in the study.

A total of 103 patients were considered eligible for inclusion in this study. In total, 25 patients were excluded for the following reasons: presence of large lung consolidation areas (n = 6), presence of diffuse interstitial involvement (n = 7), presence of macroscopic proximal pulmonary emboli (n = 8), inadequate DECT scan (n = 4). We enrolled 78 patients (45M; 58%), with a mean age of 70 years (SD 15 years, range 24–97 years). The study workflow is reported in Figure 1. Clinical data of the patient population are reported in Table 1.

In our cohort, 16.7% had a good outcome (11.5% discharged from emergency room without symptoms, 5.1% discharged from emergency room with mild symptoms) while 83.3% had a bad outcome (68.0% hospitalized in a general care unit, 10.3% hospitalized in an intensive care unit, 5.1% deceased).

Among those who were hospitalized, the mean time of hospitalization was 16.3 days (SD 10.8 days).

The mean absolute pulmonary volume was 3858.6 mL (± 1308.1 mL). Overall, the mean absolute lung enhancement was 30.7 HU (± 7.7 HU). We noticed that 16 patients (4.9%) had values of perfusion under the 25th percentile of absolute enhancement value, suggesting the presence of microscopic pulmonary emboli in those patients.

**Figure 2:**
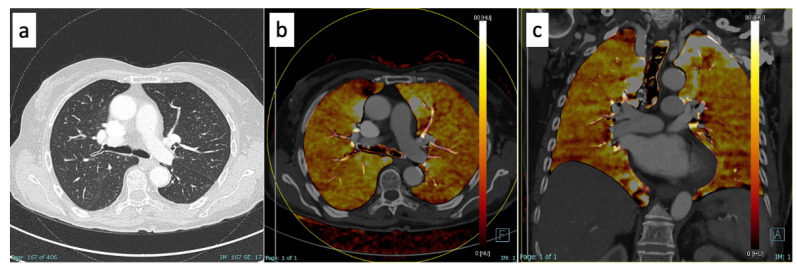
Images of a 56-year-old man diagnosed with COVID-19 via PCR analysis of an oropharyngeal swab when he entered the emergency room with shortness of breath. He underwent DECT with contrast media for the suspicion of embolic disease, which was not confirmed. On the lung window of axial DECT (**a**) there were no significant consolidations or interstitial thickening. Upon DECT reconstruction with “lung analysis” software in axial (**b**) and coronal planes (**c**), there was no evidence of hypo- or hyperperfusion. He needed hospitalization in a general care unit for ten days (bad outcome sub-group). DECT = dual-energy CT.

**Figure 3:**
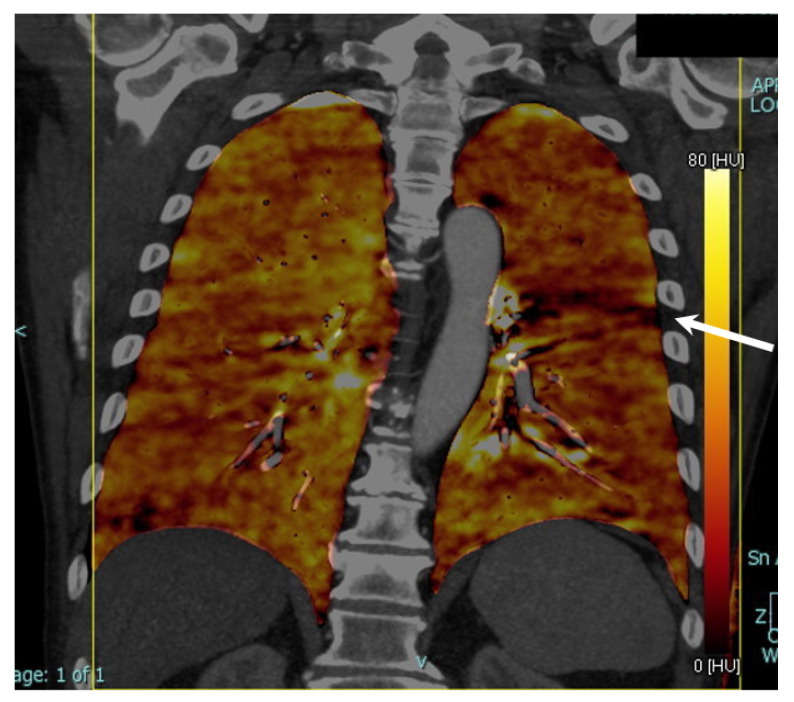
Images of a 68-year-old man diagnosed with COVID-19 via PCR analysis of an oropharyngeal swab when he entered the emergency room with respiratory distress. He underwent DECT with contrast media for the suspicion of embolic disease, which was not found. Upon DECT reconstruction with “lung analysis” software in coronal plane there was an area of hypoperfusion in the upper-middle segmentation of the left lobe (arrow). He needed hospitalization in a general care unit for five days (bad outcome sub-group). DECT = dual-energy CT.

Figure 2 shows an example of normal perfusion in a COVID-19+ patient of the bad outcome sub-group, while Figure 3 shows a case of hypoperfusion of the upper-middle segmentation of the left lobe in a patient of the bad outcome sub-group.

Total lung volume, absolute enhancement and relative enhancement parameters in the two subgroups of patients are summarized in Table 2. The total lung volume showed a statistically significant difference (*p* = 0.0116, mean difference of 684 mL) between patients with a good (mean volume 4252 ± 229.0 mL) and a bad outcome (3578 ± 185.1 mL)On the contrary, the absolute enhancement did not differ significantly between the two subgroups, being 29.8 ± 7.2 HU for patients with a good outcome and 31.2 ± 8.1 HU for patients with a bad outcome (*p* = 0.7824). Similarly, the relative enhancement did not differ significantly between patients with a good outcome (124.2 ± 65.0 HU) and those with a bad outcome (113.1 ± 33.3 HU), *p* = 0.1984.

Volumes and enhancement parameters are displayed in Table 3 and Table 4, respectively.

Interestingly, a statistically significant difference (*p* = 0.0116) was determined comparing the mean total lung volume in the subgroup with a bad outcome (3577.8 mL, ±185.1) to that of the subgroup with a good outcome (4262 mL, ±229.0).

In the good outcome sub-group, the mean absolute enhancement was 29.8 HU (±7.2 HU), while among those patients with a bad outcome, it was 31.2 HU (±8.1 HU). According to these results, we did not observe a statistically significant difference (*p* = 0.679) as regards absolute enhancement between the subgroups.

A similar result was obtained as regards relative enhancement (*p* = 0.918). In fact, in the good outcome subgroup, the mean relative enhancement was 124.2% (±65.0%), while among those with a bad outcome, it was 113.1% (±33.3%).

We had no cases with focal hypoperfusion because we excluded a priori macroscopic embolic thrombi.

In the multivariate model, all the analyzed parameters (age, sex, hospitalization time, total lung volume, vessels enhancement, total enhancement, relative enhancement) were not correlated with the presence of COVID-19. Clinical, lung perfusion and lung volume data are summarized in Table 1, Table 2, Table 3 and Table 4.

## 4. Discussion

In this paper, by using contrast-enhanced DECT, we evaluated COVID-19+ patients that presented to the emergency ward because of mild respiratory symptoms and we quantitatively assessed and compared the pulmonary volumes and absolute and relative lung enhancement.

Given the increasing evidence supporting the role of vascular pathology in the underlying pathophysiology of COVID-19 + patients, we tried to find out if a reduced vascularization could be associated with the presence of severe infection or a bad clinical outcome. For this purpose, we performed a quantitative assessment of pulmonary volumes and enhancement, excluding patients suffering from large consolidation areas, a diffuse interstitial involvement or a proximal pulmonary embolism to avoid selection bias.

To our knowledge, our study is the first to consider lung enhancements and pulmonary volumes in a population with a homogeneous (early) stage of COVID-19.

Our results did not show a significant drop in the enhancement values for COVID-19+ patients with a bad outcome, compared to the good outcome subgroup.

A possible explanation is that, in the early phases, subtle perfusion changes could be missed by the scanner. In addition, the coexisting hypoperfused and hyperperfused areas, as suggested by previous studies, even if not directly identifiable at the qualitative assessment of lung perfusion images, could balance each other, leaving a normal pulmonary enhancement.

Conversely, COVID-19+ patients with bad outcomes showed a significant drop in average pulmonary volumes (*p* = 0.0116) in comparison with patients of the good outcome subgroup. The lower lung volumes could be explained by the reduction of the pulmonary expansibility, caused by the inflammation of the interstitial involvement, with subsequent reduced elasticity and compliance. A few studies demonstrated that COVID-19 patients have a significantly reduced lung volume with increased density and mass [23,24]. In particular, Iwasawa et al. found that secondary lobes in the crazy-paving pattern were smaller than in unaffected lungs, indicating that the local volume loss was probably due to an alveolar collapse [24]. Another study by Shi et al. compared the lung volumes of COVID-19+ patients with those of non-COVID-19 patients and they found that COVID-19+ patients had a significantly reduced lung volume with increased density and mass [23].

Several studies have analyzed the role of DECT in the identification of pulmonary perfusion patterns in COVID-19 pneumonia, with inconsistent results. Ridge et al. reported a significantly decreased (qualitative and quantitative) lung enhancement on dual-energy CT pulmonary angiography in patients with severe COVID-19 pneumonia after fourteen days [26]. In the above-mentioned study, however, twenty-seven consecutive patients with acute phase COVID-19 pneumonia and severe respiratory failure were enrolled. Moreover, all the included patients were mechanically ventilated. This result is in contrast with our study, probably because we evaluated early-stage COVID-19+ patients, excluding those cases with large consolidation areas or advanced interstitial disease. This observation was also confirmed by Si-Mohamed et al. and Idilman et al., who found lobes with predominant ground glass opacities (early phase of disease) to be hyperperfused and lobes with predominant consolidation (late phase of disease) to be hypoperfused [7,27]. Conversely, Grillet et al. found hyperperfusion of both ground-glass opacities and consolidation [28]. Lang et al. and Poschenrieder et al. reported a mixture of hypo- and hyperperfused opacifications in patients with severe COVID-19 pneumonia [8,16]. Arru et al. found that ground-glass opacities demonstrated increased iodine distribution, while mixed and consolidative opacities had reduced iodine on dual-source DECTA but increased or heterogeneous iodine content on single-source DECT angiography [29].

Most of the studies performed so far, however, included patients with severe consolidation or ground-glass opacities, while we considered only cases with an early-stage disease and without a severe lung involvement. According to our hypothesis, the apparently contrasting results of the other studies can be explained because of the different phases of infection. Indeed, a similar consolidation area could be imaged in the acute phase of disease, associated with severe inflammation changes and hyperperfusion of adjacent tissues, but also in a chronic phase with a predominantly reparative phase with scarring and endovascular thrombosis phenomena, yielding to hypoperfusion. In addition, the authors used a qualitative assessment of perfusion abnormalities, while we performed only a quantitative evaluation of lung volumes and perfusion values in the hypothesis that they could show a subtle change of the pulmonary microcirculation and, possibly, the hypoperfusion changes related to micro-embolism phenomena that affect patients with COVID-19. As with the quantitative evaluation, visually we did not notice significative changes in the affected zone with respect of the surroundings areas.

In a study that investigated the relationship between lung perfusion in COVID-19+ and non-COVID-19 pathologies, Brendlin et al. used artificial intelligence to differentiate COVID-19 from immunotherapy-related pneumonitis, and they detected that COVID-19 had a significantly higher iodine uptake and concentration per pulmonary lobe than pneumonitis [30]. Similarly, we tried to stratify the risk of clinical worsening in early-stage COVID-19+ patients. For this purpose, we divided our study population into two subgroups and compared lung volumes and perfusion parameters quantitatively.

What we found was that there was no statistically significant difference between the two subgroups, neither for absolute nor for relative enhancement, underlining the impossibility to stratify the patients and the prognosis just considering the quantitative perfusion parameters at DECT.

Additional studies with larger population are needed to examine the role of lung volumes and lung perfusion parameters. A database with standard perfusion and ventilation parameters could be obtained from a larger number of patients scanned with chest CT for other purposes (for example oncologic patients with spared lungs). These data could be used in the future to identify any lung perfusion abnormality at an early phase.

This study has some limitations. Firstly, we enrolled a small number of patients. However, in contrast to previous papers, we included only patients at early phases of infection, free from consolidation or severe interstitial involvement, to reduce the presence of biases. In addition, we excluded patients with macroscopic proximal emboli in order to exclude the missing correlation with upstream pulmonary embolism as a reason for the conflicting results. Furthermore, we could not evaluate possible CT parenchymal changes at follow up in relation to clinical outcome, because only a few patients underwent CT after 1–3 months. Finally, we did not perform a correlation between DECT and lab parameters, in fact we focused only on the role of CT lung volumes and perfusion parameters. Similarly, we could not achieve a radiological–pathological correlation that could give us the chance to clearly reveal the presence of micro-embolism and early signs of inflammation in patients with lung infection.

## 5. Conclusions

In conclusion, there were no significant differences concerning the lung enhancement parameters in COVID-19+ patients with bad outcomes versus patients with good outcomes. There were, instead, some differences in the lung volumes, possibly related to a reduction of the pulmonary compliance, caused by the inflammation of interstitial involvement.

## Figures and Tables

**Table 1:** Clinical data of the patient population. Data are presented as mean value with (range) and [SD] or with (percentage).

Parameter	Value
Patients (total)	78
Age (years)	70 (24–97) [15]
Good outcome - discharged from emergency room without symptoms - discharged with mild symptoms	13/78 (16.7%) 9/78 (11.5%) 4/78 (5.2%)
Bad outcome - hospitalized in general care unit - hospitalized in intensive care unit - deceased	65/78 (83.3%) 53/78 (68.0%) 8/78 (10.3%) 4/78 (5.1%)

**Table 2:** Comparison of total lung volume (mL), absolute enhancement (in HU) and relative enhancement (%) parameters in the two subgroups of patients. The results are presented with mean value (± standard deviation) and (confidence interval 95%).

	Good Outcome	Bad Outcome	*p* Value
Total Lung Volume (mL)	4262.1 (±1295.5) (C.I.: 3795.1–4729.2)	3577.8 (±1255.1) (C.I.: 3205.1–3950.5)	0.0116
Absolute Enhancement (hu)	29.8 (±7.2) (C.I.: 27.2–32.5)	31.2 (±8.1) (C.I.: 28.8–33.6)	0.7824
Relative Enhancement (%)	124.2 (±65.0) (C.I.: 99.5–149.0)	113.1 (±33.3) (C.I.: 103.2–123.0)	0.1984

**Table 3:** Values for lung volumes (mL) obtained by automatic segmentation in the different lobes. (The *N*. values for some parameters are less than the total number either because the automatic segmentation did not include the left middle lobe or the relative enhancement was not correctly calculated).

	*N*.	Mean	St. Dev.	Min	Max
Total Lung Volume (mL)	78	3858.5	1308.1	1197	6319
Right	78	2062.1	711.6	593	3422
Left	78	1801.4	645.2	596	3177
Right Upper	78	798.4	278.4	238	1430
Right Middle	78	495.1	242.8	118	1186
Right Lower	78	773.9	273.1	236	1419
Left Upper	78	820.8	309.1	265	1661
Left Middle	20	692.5	205.6	263	1066
Left Lower	78	808.2	304.5	261	1649

**Table 4:** Values of absolute and relative enhancement obtained by automatic segmentation in different lobes. (The *N*. values for some parameters are less than the total number either because the automatic segmentation did not include the left middle lobe or the relative enhancement was not correctly calculated).

	*N*.	Mean	St. Dev.	Min	Max
Tot Enhancement (hu)	78	30.6	7.74	17	66
Right	78	30.2	8.04	4	66
Left	78	30.2	7.52	5	54
Right Upper	78	31.5	7.20	5	47
Right Middle	78	30.0	7.60	5	54
Right Lower	78	28.4	7.37	5	54
Left Upper	78	31.0	7.56	7	54
Left Middle	19	30.2	6.45	22	45
Left Lower	78	28.7	7.85	9	54
Relative Enhancement (%)	75	117.3	48.02	44	400
Right	75	116.4	49.79	12	412
Left	75	117.1	48.25	16	386
Right Upper	75	122.9	50.99	5	406
Right Middle	75	115.4	44.84	25	355
Right Lower	75	111.3	52.41	46	454
Left Upper	75	122.5	49.85	33	403
Left Middle	17	122.9	38.91	62	192
Left Lower	75	112.3	47.34	52	366

## Data Availability

Data are contained within the article.

## Abbreviations

CT	Computed tomography
DECT	Dual-energy computed tomography
COVID-19	Coronavirus disease 2019
